# The risk of hyperglycemia associated with use of dolutegravir among adults living with HIV in Kampala, Uganda: A case-control study

**DOI:** 10.1177/09564624221129410

**Published:** 2022-10-12

**Authors:** Daphine Namara, Jeremy I Schwartz, Andrew K Tusubira, Willi McFarland, Caroline Birungi, Fred C Semitala, Martin Muddu

**Affiliations:** 1 1438 Uganda Initiative for Integrated Management of Non-Communicable Diseases (UINCD), Kampala, Uganda; 2 Department of General Internal Medicine, 12228 Yale University School of Medicine, New Haven, CT, USA; 3 San Francisco Department of Public Health, Center for Public Health Research, San Francisco, CA, USA; 4 NGO for HIV care, Makerere University Joint AIDS Program (MJAP), Kampala, Uganda

**Keywords:** Hyperglycemia, diabetes mellitus, dolutegravir, HIV, Uganda

## Abstract

Emerging evidence suggests a possible association between hyperglycemia and dolutegravir (DTG), a preferred first-line antiretroviral agent in sub-Saharan Africa (SSA). There is need for rigorous studies to validate this association in the face of increasing DTG use and burden of non-communicable diseases among people living with HIV (PLHIV). We conducted a case–control study to assess the risk of hyperglycemia associated with use of DTG among PLHIV attending Mulago ISS Clinic in Kampala. Cases had hyperglycemia while controls had no hyperglycemia as confirmed by fasting plasma glucose and oral glucose tolerance tests. Demographic, laboratory, and clinical data were collected using interviewer-administered questionnaires and medical record abstraction. Analysis compared cases and controls on DTG use prior to diagnosis of hyperglycemia while controlling for potential confounders using multivariable logistic regression. We included 204 cases and 231 controls. In multivariable analysis, patients with prior DTG use had seven times greater odds of subsequent diagnosis of hyperglycemia compared to those who had non-DTG-based regimens (adjusted odds ratio [aOR] 7.01, 95% CI 1.96–25.09). The odds of hyperglycemia also increased with age (56 years and above vs. 18–35, aOR 12.38, 95% CI 3.79–40.50) and hypertension (aOR 5.78, 95% CI 2.53–13.21). Our study demonstrates a strong association between prior DTG exposure and subsequent diagnosis of hyperglycemia. Given the benefits of DTG, wide-scale use, and the growing burden of diabetes mellitus (DM) in SSA, there is need for systematic screening for hyperglycemia and consideration of alternate regimens for those at risk for DM.

## Introduction

Increased access to anti-retroviral therapy (ART) has led to a reduction in acquired immunodeficiency syndrome (AIDS)-related deaths and an increased life expectancy of persons living with HIV (PLHIV) globally.^1,2^ However, with increasing life expectancy, PLHIV are faced with an increased risk of chronic non-communicable diseases (NCDs) such as diabetes mellitus (DM) and cardiovascular diseases (CVD).^3,4^ Additionally, use of ART has been shown to increase the risk of some NCDs among PLHIV.^5,6^ For example, the protease inhibitors (PIs) have been shown to increase the risk of DM.^7,8^

To increase the safety and effectiveness of HIV treatment, newer ART medications that are better tolerated have emerged over the past decades.^9,10^ Dolutegravir (DTG) is a relatively new ART agent that belongs to the class of integrase inhibitors. It has superior potency for suppressing HIV viral load, a higher barrier to HIV resistance, and better tolerability compared to older ART such as the non-nucleoside reverse transcriptase inhibitors (NNRTIs).^11–13^ DTG-based ART regimens are progressively replacing efavirenz(EFV) containing ART regimens, the once preferred first-line ART.^11^ Due to these benefits, the World Health Organization (WHO) recommended DTG as the preferred first- and second-line ART for all adults, adolescents and children and DTG-based ART has been adopted in many low- and middle-income countries.^14^ Following WHO’s recommendation, the Uganda Ministry of Health (MoH) in its 2018 HIV treatment guidelines recommended the rollout of DTG-based ART as the preferred first- and second-line ART in the country.^15^ By the end of 2018, over 17 countries in sub-Saharan Africa had adopted and scaled up DTG-based ART.^16^

Despite its benefits, emerging evidence from case reports, case series, and clinical trials have suggested an association between DTG and disorders of glucose metabolism such as hyperglycemia.^17–20^ A more recent cross-sectional study conducted in Zambia found that when compared to NNRTI’s, DTG-based ART was associated with double the risk of developing metabolic syndrome among PLHIV; metabolic syndrome consists of hyperglycemia, hypertension, and dyslipidemia among other parameters all of which are known to increase the risk of DM.^21^ In Uganda and throughout SSA, where DTG is being widely rolled out and there is an increasing burden of NCDs among PLHIV and the general population, there is a paucity of information on the association between use of DTG-based ART and hyperglycemia.^4^ Those who develop hyperglycemia following use of DTG-based ART may need to be considered for alternate ART regimens, such as NNRTI-based which are better tolerated in this regard.^15^ Because changing the first- and second-line ART recommendations for persons at risk for DM would have profound implications for HIV care in SSA, there is great need for rigorous studies to validate the association between DTG and hyperglycemia. To this aim, we conducted a case–control study among adult PLHIV and receiving care at Mulago Immune Suppression Syndrome (ISS) clinic in Kampala, Uganda to determine the risk of hyperglycemia associated with use of DTG-based ART.

## Methods

### Study setting

The study was conducted at Mulago ISS clinic in Kampala, the largest HIV care center in Uganda. The clinic provides comprehensive HIV services to over 16,500 adults, adolescents, and children. Mulago ISS clinic adopted the use of DTG in March 2018 following the Uganda MoH guidelines for HIV treatment and prevention. Currently more than half of the total patient population is managed with DTG-based ART.

### Study design

We conducted an unmatched case–control study to assess the risk of hyperglycemia associated with use of DTG-based ART among adult PLHIV who were receiving care from Mulago ISS clinic.

### Definition of cases and controls

True cases were defined as PLHIV aged 18 years and above who were on ART, receiving HIV care from Mulago ISS clinic, and had hyperglycemia as confirmed by fasting plasma glucose (FPG) test of ≥ 100 mg/dl or 2-h oral glucose tolerance test (OGTT) of ≥ 140 mg/dl.^22–24^ To strengthen the inference of cause before effect, we excluded all cases whose diagnosis of hyperglycemia was made or known prior to March 2018 (i.e., when DTG was introduced at the clinic). True controls were defined as PLHIV aged 18 years and above who were on ART, receiving HIV care from Mulago ISS clinic, and had normal FPG of less than 100 mg/dl or a normal 2-h OGTT of less than 140 mg/dl.^22–24^ Pregnant women were excluded from the study because they were initially not eligible for DTG use in early 2018 due to the suspected risk of teratogenicity. Although they were later declared eligible in the Uganda MoH HIV treatment guidelines of 2020, there may persist some bias against its prescription during pregnancy.^15^ Additionally, recent studies emphasize the need to be cautious when prescribing DTG to pregnant women and this may also contribute to the bias against its prescription during pregnancy.^25^

### ART regimens

ART regimens were categorized into DTG-based ART and non-DTG-based ART. DTG-based regimens were all ART regimens that contained DTG in combination with two nucleoside reverse transcriptase inhibitors (NRTIs) such as tenofovir (TDF), abacavir (ABC), or zidovudine (AZT). Non-DTG-based ART were all regimens that consisted of two NRTIs in combination with another anti-retroviral drug from the NNRTIs class such as EFV, nevirapine (NVP), or from the protease inhibitors class such as lopinavir/ritonavir (LPV/r) or atazanavir/ritonavir (ATV/r).

### Identification of cases and controls

To identify cases, we reviewed clinical and laboratory records to find patients with documented evidence of hyperglycemia or use of diabetic medication such as oral hypoglycemics or insulin. Additionally, we identified those who presented at the triage area and consultation rooms for clinicians with signs and symptoms of hyperglycemia. For every case identified, one control was randomly selected from the electronic health record at Mulago ISS clinic, and their clinical records were reviewed to rule out documented evidence of hyperglycemia.

### Recruitment of cases and controls

All identified cases and controls were approached, and those who consented to participate in the study were subjected to an FPG test and a 2-h oral OGTT to confirm them as true cases or controls. We recruited participants over a period of 2 months (February-April 2021). For efficiency of recruitment and to enable examination of other associations with hyperglycemia among PLHIV, cases and controls were not matched on age, sex, or other clinical conditions. These potential confounding factors were controlled for through multivariable regression analysis.

### Laboratory procedures

We instructed consenting participants to fast for at least 8 hours prior to the glucose tests on the following morning. An FPG test and a 2-h OGTT were done by trained personnel at the clinic according to the 2021 American Diabetes Association guidelines.^23^

### Data collection

We obtained data on socio-demographic characteristics, lifestyle, and medical history from individual participants using interviewer-administered questionnaires. We also measured and recorded participants’ blood pressure using a validated automatic blood pressure machine, height using a stadiometer, and weight using a balanced scale. We cross-checked information on the medical history, ART regimens, and dates of ART initiation by reviewing participants’ clinical records.

### Ethical approval

The study protocol was reviewed and approved by the Institutional Review Boards of The AIDS Support Organization (TASO, approval number #TASOREC/078/2022-UG-REC-009), the Uganda National Council of Science and Technology (UNCST, #HS1077ES), and Yale University (#2000029496). All procedures performed in the study that involved human participants followed the ethical standards of the institutional and/or national research committee. We obtained written informed consent from all participants.

### Statistical analysis

Our analysis entailed describing cases and controls, cross-tabulations, and estimation of bivariate and multivariable logistic regression models. We used chi-square tests to determine significant differences between cases and controls across demographic and clinical characteristics as well as on the main exposure variables of past and current DTG use. We tested for DTG’s association with hyperglycemia while adjusting for other potential associations such as age, sex, increasing BMI, and hypertension. Using bivariate logistic regression analysis, we obtained crude odds ratios (ORs) showing the strength of association between the dependent variable (hyperglycemia) and independent variables. We used multivariable logistic regression analysis to control for confounders and determine other factors associated with hyperglycemia among PLHIV. Variables that were significant in bivariate comparisons at *p* < .05, those found to be confounding in our analysis, and variables determined from literature to be associated with hyperglycemia were entered into the multivariable models to obtain adjusted estimates. Collinear variables were not included in the model. We used the Hosmer and Lemeshow’s goodness of fit and likelihood ratio tests to identify the final model. To assess the robustness of the final model, forward and backward stepwise approaches to model selection were found to converge on the same set of predictor variables. We considered an alpha of 0.05 for the level of significance and 95% confidence interval to test for the association of prior DTG use and subsequent hyperglycemia. All data analyses were conducted using STATA software version 16.

## Results

Of 473 patients identified and approached, 456 consented to participate in the study including 218 cases and 238 controls. Twenty-one (14 cases and 7 controls) did not complete confirmatory tests for hyperglycemia and were excluded, leaving 435 participants (204 cases and 231 controls) for analysis. Significant differences existed between cases and controls in the variables of education, current BMI, having hypertension as a comorbidity, previous ART regimen, and current ART regimen (Table 1). Among the 204 cases, 54 (26.5%) had hyperglycemic levels that were diagnostic of prediabetes with 150 (73.5%) being diagnostic of DM. Of note, 75 (40.1%) cases previously on DTG were changed to other ART regimens after being diagnosed with hyperglycemia.

**Table 1.** Characteristics of cases (hyperglycemia) and controls (no hyperglycemia), patients in HIV in care, Uganda, 2020–2021.

Variable	Cases (hyperglycemia)		Controls (no hyperglycemia)		*p*-value
	*n*=204	(%)	*n*=231	(%)	
**Age categories (in years)**					
18 to 35	12	(5.9)	63	(27.3)	<0.001
36 to 45	57	(27.9)	92	(39.8)	
46 to 55	90	(44.1)	59	(25.5)	
56 and above	45	(22.1)	17	(7.4)	
**Sex**					
Male	68	(33.3)	67	(29.0)	0.330
Female	136	(66.7)	164	(71.0)	
**Level of education**					
No education	28	(13.7)	22	(9.5)	0.041
Primary	89	(43.6)	130	(56.3)	
Secondary	62	(30.4)	62	(26.8)	
Tertiary	25	(12.3)	17	(7.4)	
**Most recent viral load**					0.92
Suppressed (<1000 copies/ml)	186	(91.2)	210	(90.9)	
Non-suppressed (>1000)	18	(8.8)	21	(9.1)	
**Current BMI (kg/m**^**2**^**)**					
Underweight (under 18.5)	16	(7.8)	16	(6.9)	0.009
Normal weight (18.5 to 25)	58	(28.4)	100	(43.3)	
Overweight (25 to 30)	79	(38.8)	77	(33.3)	
Obese (over 30)	51	(25.0)	38	(16.5)	
**Hypertension as comorbidity**					
Has hypertension	68	(33.3)	12	(5.2)	<0.001
No hypertension	136	(66.7)	219	(94.8)	
**Previous ART regimen (*n*=398)**^a^					
DTG based regimen	75	(40.1)	4	(1.9)	<0.001
Non-DTG based regimen	112	(59.9)	207	(98.1)	
**Current ART regimen**					
DTG based regimen	83	(40.7)	212	(91.8)	<0.001
Non-DTG based regimen	121	(59.3)	19	(8.2)	
**Total duration of ART use**					0.284
Less than 5 years	39	(19.1)	52	(22.5)	
5 to 10 years	107	(52.5)	128	(55.4)	
11 and more years	58	(28.4)	51	(22.1)	
**Duration on DTG based regimen (*n*=365)**^b^					
Less than 1 year	55	(36.0)	75	(35.4)	0.892
1 to 2 years	90	(58.8)	128	(60.4)	
3 or more years	8	(5.2)	9	(4.2)	
**History of gestational diabetes**					
Yes	0	(0)	1	(1.0)	0.157
No	1	(1.0)	0	(0)	

^a^Analysis limited to participants who had prior use of other ART regimens.

^b^Analysis limited to participants who had used DTG.

ART = antiretroviral treatment; BMI = body mass index; DTG = Dolutegravir

Table 2 presents the multivariable logistic regression analysis assessing the association of prior DTG use and hyperglycemia while adjusting for age, current BMI, hypertension as a comorbidity, total duration of ART use, and current ART regimen. Patients with prior DTG use had seven-fold greater odds of having hyperglycemia compared to those who had non-DTG based regimens as their previous ART (aOR 7.01, 95% CI 1.96–25.09). The odds of having hyperglycemia also increased with older age (56 and above vs. 18–35 years, aOR 12.38, 95% CI 3.79–40.50) and having hypertension as a comorbidity (aOR 5.78, 95% CI 2.53–13.21).

**Table 2.** Factors associated with hyperglycemia, adult patients in HIV care, Kampala, Uganda, 2020–2021.

Variable	Crude odds ratios	95% Confidence interval	Adjusted odds ratios	95% Confidence interval
**Previous ART regimen**				
Non-DTG based regimen	1.0 (ref)		1.0 (ref)	
DTG based regimen	34.65	12.35–97.23*	7.01	1.96–25.09*
**Age categories (in years)**				
18 to 35	1.0 (ref)		1.0 (ref)	
36 to 45	3.25	1.61–6.55*	3.91	1.51–10.14*
46 to 55	8.01	3.98–16.11*	9.23	3.43–24.78*
56 and above	13.90	6.05–31.94*	12.38	3.79–40.50*
**Current BMI (kg/m^2^)**				
Underweight (under 18.5)	1.0 (ref)		1.0 (ref)	
Normal weight (18.5 to 25)	0.58	0.27–1.25	0.24	0.77–0.74*
Overweight (25 to 30)	1.03	0.48–2.19	0.59	0.20–1.76
Obese (over 30)	1.34	0.59–3.02	0.75	0.24–2.39
**Hypertension as a comorbidity**				
No hypertension	1.0 (ref)		1.0 (ref)	
Has hypertension	9.13	4.76–17.48*	5.78	2.53–13.21*
**Total duration of ART use**				
Less than 5 years	1.0 (ref)		1.0 (ref)	
5 to 10 years	1.11	0.68–1.82	0.56	0.25–1.26
11 and more years	1.52	0.87–2.66	0.68	0.28–1.66
**Current ART regimen**				
Non-DTG based regimen	1.0 (ref)		1.0 (ref)	
DTG based regimen	00.06	0.04–0.11	0.07	0.03–0.16*

*p-value < 0.05

## Discussion

Our findings in a large HIV care center in an African setting corroborate evidence from prior case reports, case series, and randomised controlled trials that have suggested an association between DTG and deranged glucose metabolism.^18–21,26,27^ The mechanism by which DTG causes deranged glucose metabolism is not well understood, although it is hypothesized that DTG causes insulin resistance by chelating magnesium ions which are needed as cofactors for effective action of insulin.^28^

Our findings indicate that PLHIV who had previously been prescribed DTG-based ART had seven times greater odds of having hyperglycemia compared to those who had been previously prescribed non-DTG ART regimens, even after controlling for potential confounders such as age, BMI, and hypertension as a co-morbidity. Notably, over 70% of the cases in our study had glucose levels that were diagnostic of DM. DM presents a great challenge to Uganda’s health care system, especially in HIV care clinics that remain underprepared to diagnose and manage DM, as is the case in other low-income countries.^29^ Based on unpublished case reports of hyperglycemia among PLHIV from various HIV care clinics in Uganda following initiation of DTG, the Uganda MoH recommended that all patients diagnosed with, or with risk factors for, DM should not be initiated on DTG. Those who develop DM while on DTG are also to be changed to alternate ART regimens.^15^ Our study findings collaborate this local evidence that these policy changes were justified. The timely change in policy also likely explains why many cases in this study were not continuing to use DTG-based ART; over 40% of the cases had been discontinued from DTG following the diagnosis of hyperglycemia.

The high number of hyperglycemia cases identified in our study indicates that gaps remain in baseline screening of patients for hyperglycemia prior to use of DTG but also the lack of consistent monitoring for those already using DTG. We used an FPG test and a 2-h OGTT to confirm the glycemic states of our study participants including those whose glucose levels qualified them as pre-diabetic. These assays are more reliable than random and fasting blood glucose tests often used in HIV care facilities in Uganda.^30^ Stakeholders in HIV service delivery should consider implementing the use of more reliable tests for hyperglycemia screening prior to initiation of DTG.

Consistent with prior studies,^31^ we also found that older PLHIV (particularly above 56 years) and those with hypertension were more likely to be diagnosed with hyperglycemia compared to younger patients and those without hypertension. Although BMI was not significantly associated with the occurrence of hyperglycemia in multivariable regression analysis controlling for other factors such as age and hypertension as a comorbidity; in bivariate analysis, PLHIV who were currently obese, or overweight were more likely to have hyperglycemia compared to those who had normal current BMI. These findings emphasize the need to screen these high-risk groups for hyperglycemia and hypertension vigilantly and consistently prior to use of DTG and to improve access to routine blood glucose monitoring for patients while on DTG.

As with all case–control studies, causal interpretation of our results is limited compared to prospective studies and clinical trials. While we classified exposure as the use of DTG prior to the diagnosis of hyperglycemia (i.e., cause before effect), it is not certain if underlying hyperglycemia existed prior to initiating DTG in all cases. It is also possible that DTG may accelerate a person to develop hyperglycemia when they have an underlying disposition, a factor we are unable to conclusively speak to in our case–control study. Due to the retrospective nature of our study, and a lack of reliable medical records, we were limited in establishing whether hyperglycemia resolved after switching from DTG-with or without treatment for hyperglycemia. Additionally, due to this same limitation of retrospective nature of our study, we were unable to establish changes in weight in relation to DTG exposure for cases and controls. However, we acknowledge that weight gain has been associated with use of DTG-based ART and consequently to an increased risk of deranged glucose metabolism and type II DM among PLHIV.^19^ Additionally, the duration of DTG exposure prior to onset of hyperglycemia could not be accurately measured to determine a dose-response effect. We also acknowledge the limitation of incomplete control for confounders both measured and unmeasured as inherent in the case-control design.

Despite the limitations in deducing the precise timing of events, our study noted a sequence of cases having no prior history of hyperglycemia while using non-DTG-based ART, being switched to DTG-based ART following the national roll out of DTG, and then taking them off DTG after finding hyperglycemia. This sequence provides insights on a possible causal association. In the absence of local trials or data from prospective cohort studies, our retrospective approach, coupled with evidence from other settings, provides an efficient mechanism to validate proposed changes to local guidelines in ART regimens.

In conclusion, our study demonstrates a strong association between prior DTG exposure and the development of hyperglycemia. Given the great benefits of DTG, the wide-scale roll-out in SSA countries, and the growing burden of DM in these settings, our data weigh in favor of systematic screening for hyperglycemia prior to initiating DTG, routine glucose monitoring, and the consideration of alternate regimens for those at risk for DM.
